# Buruli Ulcer in Long-Term Traveler to Senegal

**DOI:** 10.3201/eid1501.080123

**Published:** 2009-01

**Authors:** Khaled Ezzedine, Thierry Pistone, Jane Cottin, Laurent Marsollier, Véronique Guir, Denis Malvy

**Affiliations:** Centre Hospitalier Université St-André, Bordeaux, France (T. Pistone, K. Ezzedine, D. Malvy); Centre Hospitalier Université Angers, Angers, France (J. Cottin, L. Marsollier); Centre Hospitalier Saint-Nazaire, Saint-Nazaire, France (V. Guir); 1 These authors contributed equally to this article.

**Keywords:** Buruli ulcer, Mycobacterium ulcerans, Senegal, traveler, letter

**To the Editor:** Buruli ulcer (BU) is caused by infection of subcutaneous fat with the environmental pathogen *Mycobacterium ulcerans*. BU has been reported or suspected in more than 30 countries. It has never been reported in Senegal and Guinea-Bissau (*1*). We report a case of travel-associated BU in a French traveler to Senegal.

The patient was a 24-year-old Caucasian man who came to the University Hospital of Bordeaux, France, with a nonhealing lesion on the anterior left leg that had been present for ≈12 weeks. The patient had traveled in Senegal to the border of Guinea-Bissau from September 2006 through August 2007. His trip had begun in Dakar and proceeded south to the districts of Kaolack, Toubacouta, and Casamance. The patient stayed in Casamance during the rainy season from June 2007 through August 2007. He had been working on construction of wood dugouts, had been bare-legged regularly, and had been in contact with stagnant water.

He first noticed a lesion during June 2007, which had gradually increased to a small, centrally crusted ulcer. By the end of August 2007 (week 8 of the lesion), skin examination showed a 3 × 6-cm necrotizing ulcer with central crusting and an erythematous border (Figure). The lesion was not warm or tender but generated a seropurulent discharge. Concurrently, palpable left inguinal lymph nodes were observed.

**Figure:**
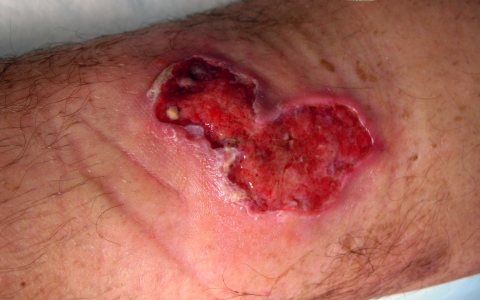
Ulcer (3 × 6 cm) on anterior side of the left leg of the patient, showing an erythematous border.

Bacteriologic swabs identified *Staphylococcus aureus* and group A *Streptoccocus pyogenes*. Two punch-biopsy specimens were taken from the border of the lesion. Histologic analysis showed nonspecific acute and chronic dermal inflammation with necrotizing granulomas that extended into the subcutaneous tissues, suggestive of infection with atypical *Mycobacterium* spp. Bacteriologic examination did not identify acid-fast bacilli (negative direct smear result after Ziehl-Neelsen staining) or other specific microorganisms (negative direct smear results after periodic acid–Schiff, Giemsa, and Gram staining). Tissue specimens were placed into BACTEC 12B broth (Becton Dickinson, Franklin Lakes, NJ, USA) (incubated at 35°C) and onto Löwenstein-Jensen slants (incubated at 30°C). No growth was detected after 42 days. On the basis of clinical findings, we suspected a diagnosis of BU.

Taq-Man real-time quantitative PCR that used primers for 2 *M*. *ulcerans*–specific genes (insertion sequence *2404* and ketoreductase B gene) (*2*,*3*) and negative controls showed positive results for DNA from both biopsy specimens. A normalized standard curve was constructed, which indicated a bacterial load of ≈6 × 10^3^ organisms/g of tissue.

Laboratory investigations indicated a total leukocyte count of 16,400 cells/μL (reference range 3,600–10,000 cells/μL) and a C-reactive protein level of 0.59 mg/mL (reference value <0.01 mg/mL). Results of radiologic investigations were normal. The patient was treated with rifampin (600 mg/day) and moxifloxacin (400 mg/day) for 12 weeks. Additional surgical excision was planned 4 weeks after treatment was begun. Unfortunately, 15 days later, the patient was lost to follow-up.

BU has been reported in many West African countries, with Guinea being the northern limit of reported cases. Detection of this case of BU suggests that the region in West Africa endemic for this disease has been underestimated or is expanding. Infection in the traveler may have occurred in Casamance, if one assumes an incubation period of 6 weeks to 3 months. Further cases should be actively sought in this region and adjoining districts visited to evaluate the geographic extent of the disease.

The environmental reservoir and mode of transmission of BU in our patient are unknown. Exposure of unprotected skin with stagnant or slow-flowing water is linked with BU. Our patient reported prolonged contact with water during his occupation. Recent studies implicating aquatic predator insects (*4**,**5*) and mosquitoes (*6*) in transmission of BU suggest that use of insect repellents and protective clothing may help prevent infection.

The diagnosis of BU in this patient relied on the PCR detection of 2 *M*. *ulcerans*–specific genes; this procedure is considered adequate (*7**,**8*). The relatively low number of organisms detected may explain the negative acid-fast bacilli smear and culture results (*9*). Our report of *M*. *ulcerans* infection from Senegal is not surprising because southern Senegal shares similar ecologic features with neighboring affected countries, especially during the heavy rainy season.

Although BU is a disease that affects mainly persons in recognized disease-endemic areas, this case emphasizes that tropical skin ulcers should be considered in differential diagnosis of BU in travelers returning from disease-endemic countries (*1*,*10*). Diagnostic delays can be avoided by use of *M*. *ulcerans*–specific PCR, a test available from World Health Organization collaborating laboratories, which enables rapid confirmation of diagnosis of BU.

## References

[1] World Health Organization. Buruli ulcer disease. *Mycobacterium ulcerans* infection: an overview of reported cases globally. Wkly Epidemiol Rec. 2004;79:194–200.15160612

[2] Fyfe JA, Lavender CJ, Johnson PD, Globan M, Sievers A, Azuolas J, Development and application of two multiplex real-time PCR assays for the detection of *Mycobacterium ulcerans* in clinical and environmental samples. Appl Environ Microbiol. 2007;73:4733–40. 10.1128/AEM.02971-06 17526786 PMC1951036

[3] Rondini S, Mensah-Quainoo E, Troll H, Bodmer T, Pluschke G. Development and application of real-time PCR assay for quantification of *Mycobacterium ulcerans* DNA. J Clin Microbiol. 2003;41:4231–7. 10.1128/JCM.41.9.4231-4237.2003 12958250 PMC193839

[4] Marsollier L, Robert R, Aubry J, Saint André JP, Kouakou H, Legras P, Aquatic insects as a vector for *Mycobacterium ulcerans.* Appl Environ Microbiol. 2002;68:4623–8. 10.1128/AEM.68.9.4623-4628.2002 12200321 PMC124085

[5] Portaels F, Meyers WM, Ablordey A, Castro AG, Chemlal K, de Rijk P, First cultivation and characterization of *Mycobacterium ulcerans* from the environment. PLoS Negl Trop Dis. 2008;2:e178. 10.1371/journal.pntd.0000178 18365032 PMC2268003

[6] Johnson PD, Azuolas J, Lavender CJ, Wishart E, Stinear TP, Hayman JA, *Mycobacterium ulcerans* in mosquitoes captured during outbreak of Buruli ulcer, southeastern Australia. Emerg Infect Dis. 2007;13:1653–60. 18217547 10.3201/eid1311.061369 PMC3375796

[7] Buruli ulcer: progress report, 2004–2008. Wkly Epidemiol Rec. 2008;83:145–54. 18437758

[8] Johnson PD, Hayman JA, Quek TY, Fyfe JA, Jenkin GA, Buntine JA, *Mycobacterium ulcerans* Study Team. Consensus recommendations for the diagnosis, treatment and control of *Mycobacterium ulcerans* infection (Bairnsdale or Buruli ulcer) in Victoria, Australia. Med J Aust. 2007;186:64–8. 17223765 10.5694/j.1326-5377.2007.tb00802.x

[9] Marsollier L, Prévot G, Honoré N, Legras P, Manceau AL, Payan C, Susceptibility of *Mycobacterium ulcerans* to a combination of amikacin/rifampicin. Int J Antimicrob Agents. 2003;22:562–6. 10.1016/S0924-8579(03)00240-1 14659652

[10] Semret M, Koromihis G, MacLean JD, Libman M, Ward B. *Mycobacterium ulcerans* infection (Buruli ulcer): first reported case in a traveler. Am J Trop Med Hyg. 1999;61:689–93. 10586895 10.4269/ajtmh.1999.61.689

